# Genotyping Characterization of *Toxoplasma gondii* in Cattle, Sheep, Goats and Swine from the North of Portugal

**Published:** 2015

**Authors:** Ana Patrícia LOPES, Anabela VILARES, Francisco NETO, Alcina RODRIGUES, Tânia MARTINS, Idalina FERREIRA, Maria João GARGATÉ, Manuela RODRIGUES, Luís CARDOSO

**Affiliations:** 1 *Department of Veterinary Sciences, School of Agrarian and Veterinary Sciences, University of Trás-os-Montes e Alto Douro (UTAD), Vila Real, Portugal*; 2 *Animal and Veterinary Research Centre (CECAV), University of Trás-os-Montes e Alto Douro (UTAD), Vila Real, Portugal*; 3 *Department of Infectious Diseases, National Institute of Health, Lisbon, Portugal*; 4 *Veterinary Services Directorate of North Region, Veterinary General-Directorate, Ministry of Agriculture, Portugal*; 5 *Parasite Disease Group, Instituto de Biologia Molecular e Celular (IBMC), Universidade do Porto, Portugal*

**Keywords:** Genotypes, Portugal, Ruminants, Swine, *Toxoplasma gondii*

## Abstract

***Background:*** Epidemiological investigations on *Toxoplasma gondii* infection have found a significant association between human toxoplasmosis and consumption of raw or undercooked meat. The present study aimed to characterize genotypes of *T. gondii* in 20 cattle, 40 sheep, 15 goats and 16 pigs from the North of Portugal.

Methods: Nested PCR amplified the surface antigen 2 (SAG2) gene. Sequencing analysis was performed in order to assess the prevalence of SAG2 type strains (I, II and III).

**Results:** Three and 4 strains of SAG2 type II were identified in heart samples of cattle and sheep, respectively. Three SAG2 type II strains were detected in brain, diaphragm and heart of 3 pigs. Three strains detected in heart samples of 3 goats belonged to SAG2 types I or II; with the same result being observed in heart samples from 2 sheep and in 2 brain and 1 heart samples from 3 pigs.

**Conclusion:** SAG2 type II has been shown for the first time to infect cattle in North of Portugal. In addition, SAG2 type II has also been confirmed as the predominant strain in sheep and pigs in the same region. This is the first molecular report of *T. gondii* in goats from Portugal.

## Introduction

Toxoplasmosis, caused by the protozoan *Toxoplasma gondii*, is a global parasitic zoonosis. Domestic and wild felids are the definitive hosts of the parasite and a great variety of other homeothermic animals are intermediate hosts. Herbivores acquire infection mainly by the ingestion of oocysts in water or contaminated food. Carnivores and omnivores, including human beings, can additionally become infected by ingesting meat with cysts (bradyzoites) or even pseudocysts (tachyzoites) (1).

Epidemiological investigations have found a significant association between human toxoplasmosis and the consumption of raw or undercooked meat or its products (1). Infections with the parasite are also an important cause of foetal mortality in sheep and goats (). It has originated outbreaks of abortion in small ruminant farms, leading to serious economic and reproductive losses (). Furthermore, *T. gondii* may affect the swine reproductive outcome as well, causing abortion or stillbirths ().

Most of the identified strains of *T. gondii* obtained from humans and animals in Europe, using polymerase chain reaction-restriction fragment length polymorphism (PCR-RFLP) and microsatellite analysis, are grouped into one of three clonal lineages of type I, II or III (). These strains have been described in Portugal, with types II and III as common in pigs and chicken (6–8). Studies in other European countries, including France, Spain, Switzerland, and Germany, have revealed a predominance of genotype II in domestic animals (9–12).

Due to the relatively scarce information on the molecular detection and characterization of *T. gondii* in animals for human consumption in Portugal, this study aimed at detecting parasitic DNA in tissues from cattle, sheep, goats and pigs raised and slaughtered in the North of the country and to genetically characterize infecting strains of *T. gondii*.

## Materials and Methods

***Animals and samples***

Between March 2008 and March 2010, heart tissue samples were obtained from cattle (n = 20), sheep (n = 40) and goats (n = 15), slaughtered in abattoirs from the North of Portugal. Heart (n = 4), brain (n = 10), diaphragm (n = 6) and tongue (n = 1) tissues were randomly sampled from 16 slaughtered pigs. All the sampled animals had been born and raised in the North of Portugal and were intended for human consumption. Individual tissue samples were stored at -20 ºC until DNA extraction. Data were collected for each sampled animal included gender, breed, age in months and husbandry system (intensive, semi-intensive or extensive). Twelve cattle, 34 sheep, 5 goats and the 16 pigs were seropositive to *T. gondii*, as determined by an antibody titer ≥ 20 in the modified agglutination test (13).

This study was approved by the UTAD Veterinary Hospital Ethical Committee as complying with the Portuguese legislation for the protection of animals (Law no. 92/1995).

***DNA extraction***

Tissue samples were digested in a trypsin solution for 2 hours at 37 ºC, with pellets being stored at -20 ºC until use. DNA extraction was performed as previously described by Miller et al. (14). DNA was precipitated by addition of 100% ethanol (-20 ºC), resuspended in 300 µl of DNAse/RNase free water and stored at -20 ºC until use.

***Polymerase chain reaction***

Nested PCR separately amplified the 5’ and 3’ ends of the surface antigen 2 (SAG2) gene (15), following the specific protocol described for AmpliTaq DNA polymerase^®^ (Applied Biosystems, Foster City, USA). Two PCR were used to amplify external sequences of the first and second fragments of gene SAG2. The first fragment was amplified with primers SAG2.F4 (5’ GCT-ACC-TCG-AAC-AGG-AAC-AC 3’) and SAG2.R4 (5’ GCA-TCA-ACA-GTC-TTC-GTT-GC 3’); and the second fragment with SAG2.F3 (5’ TCT-GTT-CTC-CGA-AGT-GAC-TCC 3’) and SAG2.R3 (5’ TCA-AAG-CGT-GCA-TTA-TCG-C 3’). A reaction mixture was used comprising 10 µl of extracted DNA, PCR buffer [2 mM MgCl_2_, 50 mM KCl, 10 mM Tris-HCl (pH 8.3)], 0.25 mM dATP, 0.25 mM dGTP, 0.25 dCTP, 0.25 dTTP, 1.25 U AmpliTaq DNA polymerase^®^, 20 ρmol of each external primer, and DNAse/RNAse free water to a final volume of 50 µl. PCR amplification was performed under the following conditions: 95 ºC for 10 min; 40 cycles of 94 ºC for 45 s; 53 ºC for 45 s; 72 ºC for 90 s; and then final at 72 ºC for 10 min.

One microliter of the initial PCR products was used for amplification of internal sequences of gene SAG2 first and second fragments. The first fragment was amplified with primers SAG2.F (5' GAA-ATG-TTT-CAG-GTT-GCT-GC 3') SAG2.R2 (5' GCA-AGA-GCG-AAC-TTG-AAC-AC 3'); and the second fragment with SAG2.F2 (5' ATT-CTC-ATG-CCT-CCG-CTT-C 3') and SAG2.R (5' AAC-GTT-TCA-CGA-AGG-CAC-AC 3'). Reagent concentrations and reaction conditions were the same as described above. For both amplifications, PCR mixtures with a negative result were diluted at 1:10 and retested.

Positive (*T. gondii* RH strain DNA) and negative (DNAse/RNAse free water or AmpliTaq DNA polymerase^®^ buffer) controls were included in every amplification batches. All amplification products were analyzed by a 2% agarose gel electrophoresis with soaking GelRed^TM^ dye (Biotium, Hayward, USA). A molecular marker was used in all assays (φ X174/RF DNA/Hae III Fragment; Invitrogen, Grand Island, USA). Visualization of a 241 bp (first fragment sequence) or a 221 bp band (second fragment sequence) was considered a positive result.

***Genotyping***

Sequence analysis for comparison of SAG2 genetic profiles was performed in order to assess the prevalence of *T. gondii* SAG2 type strains (I, II and III). Nested PCR products were purified with ExoSAP-IT^®^ (Affymetrix, UK) and sequenced using BigDye^TM^ (Applied Biosystems, USA), following manufacturer’s instructions and using the internal primers. Products were purified through DyeEx^®^ columns (Qiagen, Crawley, UK) and processed in an automatic sequencer 3130 XL Genetic Analyzer (Applied Biosystems, USA). Results were aligned with BioEdit program and compared to the following sequence data available from GenBank: AM055943.1 RH type I, XM002371960.1 ME49 type II, AF357579.1 NED type III, AF357582.1 CASTELLS atypic strain, and AF357580.1 MAS atypic strain (GenBank).

***Data analysis***

McNemar test compared PCR and serology results in paired samples, i.e. those obtained from the same animal. Statistical analyses were done with SPSS software for Windows (Chicago, IL, USA).

## Results

**Fig. 1:**
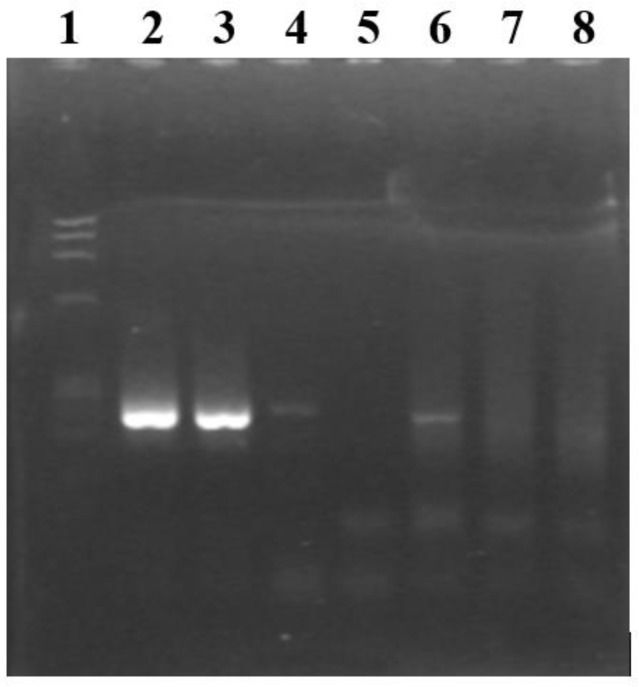
PCR (SAG2.F and SAG2.R2 primers) gel electrophoresis. Band 1: molecular weight marker; band 2: positive control (~241 bp); bands 3, 4 and 6: positive results; band 5: negative control; bands 7 and 8: negative results

Table 1 presents PCR results in the 75 ruminants (20 cattle, 40 sheep and 15 goats). Figure 1 shows gel electrophoresis after PCR amplification with SAG2.F and SAG2.R2 primers.

**Table 1:** SAG2 PCR results in randomly selected heart samples of seropositive (n = 51) and seronegative (n = 24) ruminants from the North of Portugal

**Ruminants**	**Animals (n)**	**Relative distribution (%)**	**PCR positive ** **(%)**	**McNemar ** **test**
Cattle				
Seropositive	12	60.0	50.0	
Seronegative	8	40.0	0.0	*P* = 0.031
Sheep				
Seropositive	34	85.0	17.6	
Seronegative	6	15.0	0.0	*P *< 0.001
Goats				
Seropositive	5	33.3	60.0	
Seronegative	10	66.7	0.0	*P* = 0.500
Total				
Seropositive	51	68.0	29.4	
Seronegative	24	32.0	0.0	*P* < 0.001

**Table 2:** *Toxoplasma gondii* SAG2 strains detected in cattle, sheep, goats and pigs from the North of Portugal

**Strain**	**SAG2 genotype**	**Gender**	**Breed**	**Age group**	**Husbandry system**	**MAT titer**
						
Cattle						
TgPtB_1_	II	M	Crossbreed [H]	]8-12 months]	Semi-intensive	100
TgPtB_2_	II	F	Crossbreed [H]	]8-12 months]	Semi-intensive	100
TgPtB_3_	II	M	Crossbreed [H]	]8-12 months]	Semi-intensive	100
TgPtB_4_	neg	F	Mirandesa [H]	]8-12 months]	Semi-intensive	100
TgPtB_5_	neg	M	Crossbreed [H]	[5-8 months]	Semi-intensive	100
TgPtB_6_	neg	M	Arouquesa [H]	[5-8 months]	Semi-intensive	100
Sheep						
TgPtS_1_	II	F	Cross of Churra [H]	≥ 19 months	Extensive	20
TgPtS_2_	I or II	M	Churra Terra Quente [H]	[7-18 months]	Semi-intensive	≥ 6400
TgPtS_3_	II	M	Churra Badana [H]	≤ 6 months	Semi-intensive	20
TgPtS_4_	II	M	Churra Terra Quente [H]	≤ 6 months	Semi-intensive	20
TgPtS_5_	II	M	Churra Terra Quente [H]	≤ 6 months	Semi-intensive	20
TgPtS_6_	I or II	F	Churra Terra Quente [H]	≤ 6 months	Semi-intensive	400
Goat						
TgPtG_1_	I or II	M	Preta de Montesinho [H]	≤ 6 months	Semi-intensive	20
TgPtG_2_	I or II	M	Preta de Montesinho [H]	≤ 6 months	Semi-intensive	20
TgPtG_3_	I or II	M	Preta de Montesinho [H]	≤ 6 months	Semi-intensive	20
Pig						
TgPtP_1_	I or II	M	Cross of Bísaro [H]	≤ 3 months	Intensive	400
TgPtP_2_	II	F	Cross of Bísaro [H]	≤ 3 months	Intensive	400
TgPtP_3_	II	F	Cross of Bísaro [Br]	[4-9 months]	Intensive	20
TgPtP_4_	neg	F	Piétrain [Br]	[4-9 months]	Intensive	20
TgPtP_5_	II	F	Piétrain [D]	[4-9 months]	Intensive	20
TgPtP_6_	neg	F	Piétrain [D]	≥ 10 months	Intensive	20
TgPtP_7_	I or II	F	Piétrain [Br]	≥ 10 months	Intensive	20
TgPtP_8_	I or II	F	Bísaro [Br^a^]	≥ 10 months	Semi-intensive	20
TgPtP_9_	neg	F	Bísaro [D^a^]	≥ 10 months	Semi-intensive	20

B: bovine; Br: brain; D: diaphragm; F: female; G: goat; H: heart; M: male; MAT: modified agglutination test; neg: negative; P: pig; Pt: Portugal; S: sheep; Tg: *Toxoplasma gondii*; ^a^ same individual pig.

Table 2 displays the characterization of *T. gondii* strains detected in cattle, sheep, goats and pigs from the North of Portugal.

In 6 animals (25.0%) sequencing analysis was negative after completion of several dilutions. Due to small concentration of DNA, the second fragment (3' fragment) did not amplify, and it was not possible to differentiate between SAG2 type I and type II alleles in other eight (33.3%) out of the 24 characterized isolates.

Only four (TgPtB_1_; TgPtB_2_; TgPtS_3_; TgPtS_4_) of the 24 SAG2 positive samples were found positive by real time PCR. In addition, two SAG2 negative samples were found positive after real time PCR. 

## Discussion

This work represents the first molecular study on *T. gondii* infection in goats from Portugal and in cattle from the North region of the country. In food animals from the North of Portugal for human consumption, the seroprevalence of *T. gondii* infection has been 7.5% in cattle, 33.6% in sheep, 18.5% in goats, and 9.8% in pigs (). Still in northern Portugal, a 24.4% prevalence of antibodies to *T. gondii* has been detected in women of childbearing age ().

In the present work, 50% of the seropositive cattle were also found to be positive after SAG2 nested PCR. Results obtained in other countries report the detection of the parasite in the myocardium of 10% of seropositive cattle in Argentina (18) and in meat of 38% of cattle in the United Kingdom (19). One of the major difficulties in detecting *T. gondii* DNA in tissues of cattle is the limited size of the samples. Since 100 g of beef can contain only one tissue cyst, a negative result from a smaller sample does not necessarily mean that all the sampled tissue is free from the parasite (20).

The SAG2 gene is useful for genotyping strains of *T. gondii* and characterizing isolates as one of the three types of the clonal lineages (). Nevertheless, SAG2 does not detect strains with recombinant or atypical genotypes (21). For this reason, it is appropriate for geographical areas where the clonal lineages predominate, like Europe (22–24). This is corroborated by studies in food animals from Portugal (), France () and Switzerland (), where all genotypes obtained through the analysis of SAG2 belonged to the major clonal lineages, as confirmed by microsatellite analysis. This dominance of type II strains in Portugal was also described in humans by Esteves (7).

In the present study, genetic characterization of sheep isolates from northern Portugal has identified four strains of SAG2 type II and two strains of SAG2 type I or II (Table 2). In southern Portugal, Esteves (7) found an equal proportion of genotypes of types I and II in sheep based on analysis of SAG2 and microsatellites. The information available on the isolation and genotyping of strains from goats is very scarce worldwide. In the present study *T. gondii* was detected in three out of five seropositive goats (60%). However, the sequencing results were inconclusive, and the strains may belong to either a SAG2 type I or II genotype.

Genetic characterization of the swine strains identified three genotypes of SAG2 type II and three of SAG2 type I or II (Table 2). Also from pigs of northern Portugal, through SAG2 and microsatellites analysis, Sousa et al. (8) detected *T. gondii* in 40.5% of heart and/or brain of seropositive animals with a predominance of type II strains and fewer of type III. The isolation of the parasite is difficult or even impossible in pigs with low antibody titers (25, 26). Nevertheless, it is likely that a seropositive pig has infective forms in its tissues, and the consumption of undercooked meat or meat products from these animals is a risk factor for transmission to humans (). Furthermore, the existence of seropositivity is an indicator of the hygienic status and the risk of infection for a given pig farm (27).

In the present study, the analysis of SAG2 indicated the predominance of type II strains in animals in the North of Portugal, which may be due to increased circulation of this type in the region, but also because these strains have a greater potential of cyst formation in the host. The intermediate virulence of type II does not lead to a frequent death of the host, allowing the animals to survive until slaughtering (28). In eight cases, it was not possible to differentiate between types I and II by sequence analysis and in six other samples the results were negative probably due to a low DNA concentration.

Two samples negative for SAG2 (nested PCR) were found positive after real time PCR, and only four of the 24 positive SAG2 samples were found positive by real time PCR. 

This might be explained by the different protocols and sensitivities of the two PCR techniques. Although seroprevalence values of infection in animals are usually higher than those of the molecular analysis, there is usually a good correlation between antibody titres and the presence of viable cysts (29, ). In the present study, for cases in which PCR was negative but seroconversion has occurred the PCR result may reflect a low number or a transient presence of *T. gondii* in the sample ().

Even though in Europe SAG2 type II strains have frequently been found equivalent to the clonal type II (as confirmed by microsatellite analysis), recent data show that even in Europe there may be recombinant or atypical strains. Therefore, the present work is a preliminary study that should be complement with other investigations markers to confirm the membership to clonal type II.

## Conclusion

The present study provides a deeper knowledge on the genotypes of *T. gondii* that circulate in northern Portugal by showing for the first time SAG2 type II to infect cattle in the North of Portugal and goats in the whole country. SAG2 type II has also been confirmed as the predominant one in sheep and pigs in northern Portugal. Due to the zoonotic character of *T. gondii*, control of the infection in food animals is important for consumer protection.
